# Editorial: Severe mental illnesses in children: unravelling developmental trajectories, neuropsychiatric impairments, and chronic pain

**DOI:** 10.3389/frcha.2026.1889961

**Published:** 2026-07-21

**Authors:** Elena S. Monarch, Piero Pavone

**Affiliations:** 1 Lyme and PANS Treatment Center, Hingham, MA, United States; 2 Universita Degli Studi di Catania, Catania, Italy

**Keywords:** autism, developmental psychopatholgy, neuropsychiatry, obsessive compulsive disorder (OCD), PANDAS (Pediatric autoimmune neuropsychiatric disorders associated with streptococcal infection), PANS (Pediatric acute-onset neuropsychiatric syndrome), toxoplasama gondii, treatment-resistance

Treatment-resistant neuropsychiatric patients and their families often endure extraordinary suffering, impaired functioning, and financial burden. One of the most important lessons emerging from modern neuropsychiatry is that psychiatric symptoms, particularly in children, can sometimes represent the downstream effects of hidden neurological, immunological, gastrointestinal, structural, or environmental pathology rather than primary psychiatric disease. A growing body of research suggests that diagnostic overshadowing, where psychiatric labels inadvertently narrow the clinical lens, may delay identification of treatable underlying conditions and, in some cases, compromise recovery.

Psychiatrists Rosalie Greenberg, Robert Bransfield, Marion Leboyer and their colleagues have argued that psychiatry may be entering a new era in which immune dysfunction, inflammatory signaling, infectious burden, and blood-brain-barrier disruption should be considered alongside traditional psychiatric formulations (1–6, Greenberg). Their work provides an important conceptual framework for understanding why children with similar psychiatric symptoms may have profoundly different underlying pathophysiology.

The connection between infection and psychiatric dysfunction is not new. More than a century ago, clinicians recognized that syphilis could cause psychosis. In the early twentieth century, Sydenham's chorea revealed that streptococcal infections could trigger not only movement disorders, but obsessions, compulsions, mood lability, and psychosis (8, 14). By the late twentieth century, viral theories of psychiatric illness gained traction. Prenatal viral exposure was associated with increased schizophrenia risk in offspring (16), while herpes simplex virus and Epstein–Barr virus antibodies were linked to obsessive-compulsive and other psychiatric symptoms (9, 10).

Perhaps the most clinically influential work in pediatric neuroimmune psychiatry came from Swedo and colleagues, who described children with abrupt-onset obsessive-compulsive symptoms, anxiety, tics, eating restriction, urinary frequency, rage episodes, and behavioral regression following streptococcal infection (12, 13). This work led to the identification of Pediatric Autoimmune Neuropsychiatric Disorder Associated with Streptococcus Infection (PANDAS) and later the broader syndrome of PANS which includes other infectious or non-infectious sources of brain inflammation (18). For example, SARS-CoV-2 has been implicated as a potential trigger of PANS (15, 17).

The importance of identifying underlying factors is underscored by studies showing how this awareness can lead to recovery. A recent case series in this journal described three children presenting with severe treatment resistant neuropsychiatric symptoms such as disabling anxiety, obsessive-compulsive symptoms, tics, eating restriction, hallucinations, and school refusal (7). The children had previously received conventional psychiatric diagnoses and interventions with insufficient improvement. However, after identifying infectious triggers, gastrointestinal dysbiosis, immune activation, and inflammatory contributors, and addressing these with antimicrobials, nutraceuticals, probiotics, and dietary interventions, all three children experienced full recovery.

These findings are corroborated by studies showing that children with PANS frequently exhibit allergic symptoms, immune-mediated food reactions, and gastrointestinal dysfunction (22). Likewise, numerous studies indicate that children with the seemingly intractable neurodevelopmental condition, autism, exhibit elevated markers of both neuroinflammation and gastrointestinal distress [e.g. (19, 20, 29)]. In one post-mortem investigation, two-thirds of autistic individuals' brain tissue had evidence of immune-cell infiltration and immune-mediated cytotoxic damage (20). Collectively, these findings closely mirror clinicians’ and parents' observations that pediatric autism, OCD, and tic disorders often co-occur with immune reactivity, gastrointestinal distress, and inflammatory conditions. Seminal research published in *Nature* uncovered that the brain is not as “immune-privileged” as previously thought (23). In fact, it is now understood that there are structural and functional connections between the peripheral immune and central nervous systems, unveiling new potential pathological interactions that could lead to neurological or psychiatric diseases (21).

In this issue, Greenberg found that 92% of youth diagnosed with pediatric bipolar disorder demonstrated evidence of tick-borne or other treatable infectious exposures, such as Lyme disease, streptococcal infection and/or Mycoplasma pneumonia. According to Greenberg, treatment of infection along with anti-inflammatory care provided symptom resolution in many cases.

Among infectious contributors, one of the most underestimated may be the parasite, Toxoplasma gondii. Multiple meta-analyses have demonstrated significantly higher rates of T. gondii exposure among individuals with schizophrenia, bipolar disorder, OCD, and other severe psychiatric disorders than control groups [e.g. (11, 24, 25)]. Pediatric studies similarly show higher seropositivity of the parasite among children with OCD and anxiety (26). Moreover, treatment-resistant OCD patients have demonstrated higher antibody levels of the parasite (27, 28).

Other hidden drivers of mental illness may not be infectious. In this issue, Saliba and Stewart described a child presenting with severe obsessive-compulsive symptoms who was later found to have a pineal region germinoma with obstructive hydrocephalus. Following neurosurgical treatment, the child's obsessive-compulsive symptoms significantly improved.

Psychiatric outcomes can also be shaped by noninfectious systemic and environmental stressors. In this special issue, Zheng et al. found that mental health outcomes following pediatric orthopedic trauma were influenced less by injury severity and more by chronic pain, family support, and socioeconomic adversity. Similarly, in this issue, Bansema et al. demonstrated that youth with severe and enduring mental health problems are often poorly captured by symptom-based classification systems.

Taken together, these studies suggest that many childhood psychiatric presentations may simply convey common consequential behavioral pathways. Children can display similar outward symptoms despite profoundly different underlying etiologies. The future of pediatric psychiatry may depend less on refining symptom labels and more on identifying what lies beneath them.
